# The association between serum interleukin-1 beta and heparin sulphate in diabetic nephropathy patients

**DOI:** 10.1007/s10719-021-10035-7

**Published:** 2022-01-08

**Authors:** Liqiong Jiang, Jianying Zhou, Li Zhang, Yufeng Du, Mingming Jiang, Liqian Xie, Zhenni Ma, Fengling Chen

**Affiliations:** 1 Department of Nephrology, the Affiliated Suzhou Hospital of Nanjing Medical University, Suzhou, China; 2 Department of Endocrinology, the Affiliated Suzhou Hospital of Nanjing Medical University, Suzhou, China; 3 Clinical Lab, the Affiliated Suzhou Hospital of Nanjing Medical University, Suzhou, China; 4 Jiangsu Institute of Hematology, the First Affiliated Hospital of Soochow University, Suzhou, China; 5 Department of Hemodialysis Center, the First Affiliated Hospital of Soochow University, Suzhou, China

**Keywords:** Diabetic nephropathy, heparin sulphate, Interleukin 1β, Inflammasome, Endothelial glycocalyx, Microalbuminuria

## Abstract

Inflammation is considered an important mechanism in the development of diabetes mellitus (DM) and persists for a long time before the occurrence of diabetic nephropathy (DN). Many studies have demonstrated that a decrease in the endothelial glycocalyx (EG) is negatively correlated with proteinuria. To elucidate whether EG damage induced by inflammasomes in DM patients leads to the occurrence of microalbuminuria (MA) and accelerates the progression of DN, this study screened 300 diagnosed DM patients. Finally, 70 type 2 diabetes patients were invited to participate in this study and were divided into two groups: the T2DM group (patients with normal MA and without diabetic retinopathy, n = 35) and the T2DN group (patients with increased MA and diabetic retinopathy, n = 35). Circulating heparin sulphate (HS, EG biomarkers) and interleukin-1 beta (IL-1β, inflammasome biomarkers) of the patients were measured by ELISA. Laboratory data were measured using routine laboratory methods. Patients in the T2DN group had increased serum HS, increased IL-1β, increased CRP, decreased haemoglobin, and increased neutrophils compared to patients in the T2DM group (all P < 0.05). Increased HS and decreased haemoglobin were independently associated with T2DN patients. ROC curves showed that the AUC of HS for the prediction of T2DN was 0.67 (P < 0.05). The combination of HS and haemoglobin yielded a significant increasement in the AUC (0.75, P < 0.001) with optimal sensitivity (71.2%) and specificity (79%). Furthermore, serum IL-1β was positively correlated with HS and was an independent associated factor of HS in the T2DN group. The relationship between HS and IL-1β was not significant in the T2DM group. Our findings surgessed the inflammasome may be associated with and promote damage to the EG during the disease course of DN that manifests as increased MA.

**Supplementary information:**

The online version contains supplementary material available at 10.1007/s10719-021-10035-7.

## Introduction

The global diabetes mellitus (DM) prevalence in 2019 was estimated to be 9.3% (463 million people) and is expected to increase to 10.2% (578 million) by 2030 and 10.9% (700 million) by 2045 [1]. Approximately 30% to 40% of patients with DM develop diabetic nephropathy (DN) [2]. DN is the single most common cause of end-stage renal disease (ESRD) in many parts of the world [3].

Because actual loss of renal function is a late indicator of DN, albuminuria has been proposed as a sensitive surrogate marker for ongoing renal injury in DN [4]. Much evidence indicates that endothelial glycocalyx (EG) damage results in disordered microvascular permeability, which in the kidney manifests as albuminuria [5–8]. The EG is a negatively charged gel that coats the endothelium and creates a molecular sieve that prevents large molecules from passing through and likely protects endothelial cells [9]. The EG consists of glycoproteins, proteoglycans, glycosaminoglycans and associated plasma proteins, and hyaluronic acid and heparin sulphate (HS) are its major constituents [10]. The EG is very fragile and easily deteriorates [11], therefore, one possibility to investigate the state of the glycocalyx is to measure retention or shedding of its constituent parts, such as HS, syndecan-1 or hyaluronan [12–14]. There is a negative association between the EG and proteinuria [15]. Maintaining the structural integrity of EG may prevent proteinuria [16].

There is increasing evidence for the role of the inflammatory response both in developing DM and its associated complications, including DN [17]. Various molecules related to the inflammatory pathways in DN include transcription factors, proinflammatory cytokines, chemokines, adhesion molecules, Toll-like receptors, adipokines and nuclear receptors, which are candidates for new molecular targets for the treatment of DN [18]. Activation of the Nod-like receptor family pyrin domain containing 3 (NLRP3) inflammasome is directly related to an excessive inflammatory response and, therefore, is directly linked to the pathophysiology of chronic inflammatory disorders, such as DM, and its associated complications [19]. The assembly of the NLRP3 inflammasome complex creates a potent inflammatory multiprotein that can upregulate inflammatory cytokines, such as interleukin 1 beta (IL-1β) and interleukin 18 (IL-18). [19] EG damage can be triggered by exposure to pathogens, microbial toxins, or endogenous danger signals [20]. Whether the NLRP3 inflammasome damages the EG in DM and DN patients is not well investigated.

Based on these mechanisms, we hypothesized that the NLRP3 inflammasome in type 2 diabetes (T2D) patients would be associated with pathological degradation of the EG and excretion of EG fragments into the circulatory system. Damage to the EG would therefore be associated with the development of microalbuminuria (MA) and DN. We performed a prospective study of T2D patients to test this hypothesis. HS was used as a marker of EG degradation, and IL-1β was used as a marker of the NLRP3 inflammasome in this study.

## Materials and methods

### Study design and population

**Fig. 1:**
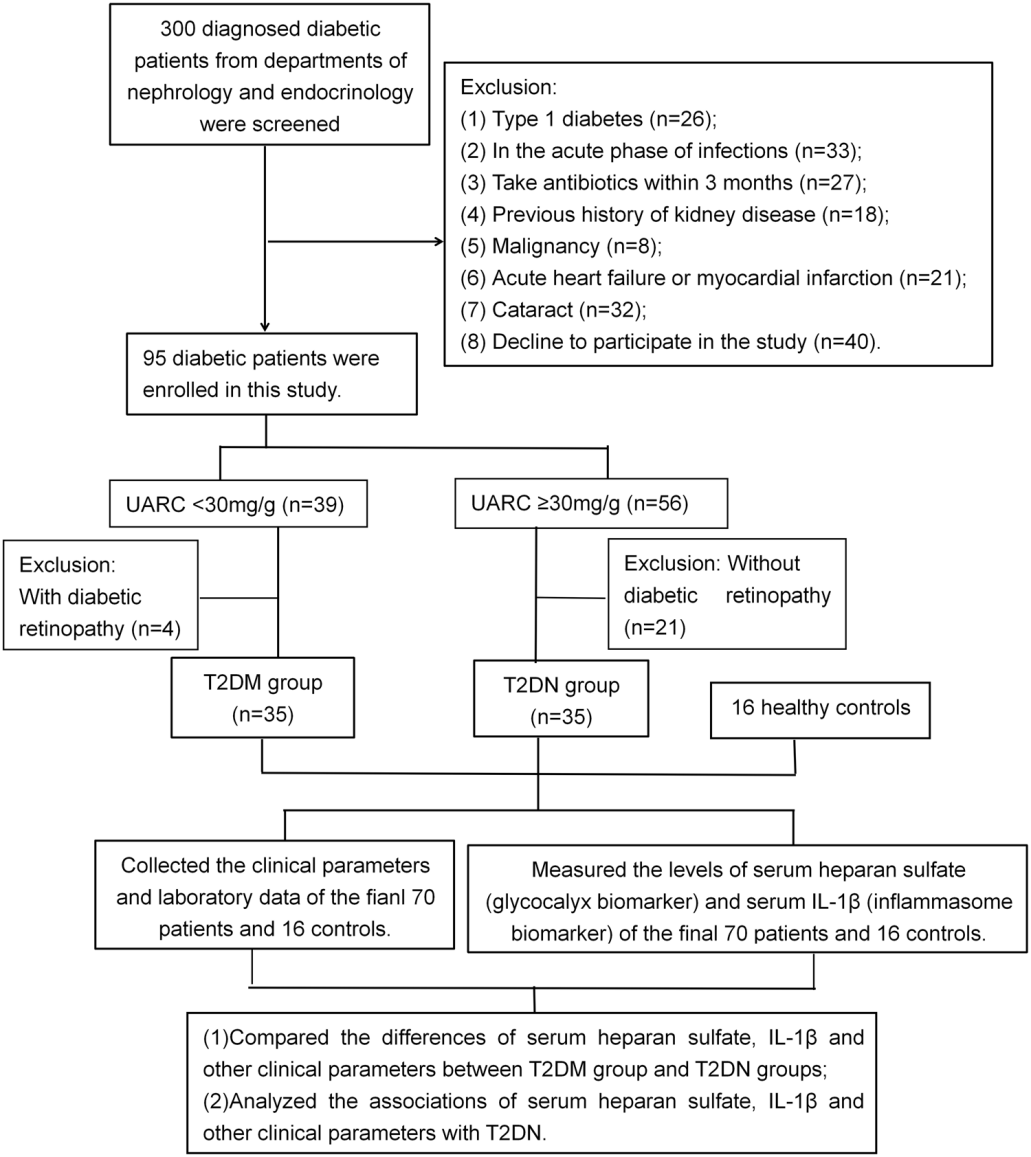
Flow chart of the prospective, observational cohort. UACR, urinary albumin-to-creatinine ratio

This cross-sectional, observational study screened 300 diagnosed diabetic patients (age > 18 years old) from the Departments of Nephrology and Endocrinology of Suzhou Municipal Hospital from May to December 2020. The exclusion criteria are described in the flow chart (Fig. 1). Finally, 70 T2D patients were invited to participate in this study. Additionally, we obtained data from 16 healthy controls from the Physical Examination Center of Suzhou Municipal Hospital. Normal MA was defined as a urinary albumin-to-creatinine ratio (UARC) less than 30 mg/g. [21] Seventy T2D patients were divided into a T2DM group (n = 35) and a T2DN group (n = 35). The T2DM group was defined as patients with normal MA (UARC < 30 mg/g) without diabetic retinopathy, and the T2DN group was defined as patients with increased MA (UARC 30 to 300 mg/g) and diabetic retinopathy. Blood samples were collected in dry tubes, centrifuged to obtain the serum and stored at -80 °C for a maximum of 6 months before the final measurement.

### Demographic characteristics and laboratory data collection

Demographic information, including age and sex, was recorded for each patient. Serum albumin, fasting blood glucose (FBG), urea nitrogen, creatinine, uric acid, carbon dioxide, triglyceride, total cholesterol, high-density lipoprotein (HDL), low-density lipoprotein (LDL), c-reactive protein (CRP), glycosylated haemoglobin (HbAlc), haemoglobin (HGB), platelet, leukocyte, neutrophil, and neutrophilic granulocyte percentage (N%) were measured using routine laboratory methods.

### Measurement of serum IL-1β and HS in diabetic patients

Serum IL-1β levels were assessed by enzyme-linked immunosorbent assay (ELISA) kits for human IL-1β (Catalogue No: KE00021, Proteintech, China). Serum HS levels were assessed by ELISA kits for human HS (Catalogue No: F01027, Westang Biotech, China). Levels of serum IL-1β and HS were measured according to the manufacturers’ instructions. Details of the measurements are provided in the Supplementary Methods.

### Statistical analyses

Continuous variables are shown as the mean ± standard error or as the median with interquartile range. Categorical variables are expressed as percentages. Univariate analyses were performed to compare differences between the T2DM and T2DN groups. Student’s t-test was used to compare normally distributed data, while the Mann–Whitney U-test was used for nonnormally distributed data. Categorical data were compared using the Chi-squared test. Comparisons of the three groups (T2DN, T2DN and healthy control groups) were performed using one-way ANOVA. Bivariate correlation analyses were performed to assess the correlation of variables. Logistic regression analyses were performed to evaluate variables independently associated with T2DN. ROC curves were generated to calculate the area under the curve (AUC) and compare the prognostic value of every independently associated factor or united factor to T2DN. Furthermore, in the T2DN group, 35 patients were divided into two groups according median of serum HS, multivariate linear regression analyses were performed to identify independent factors associated with HS. All analyses were two-tailed, and P < 0.05 was considered statistically significant. SPSS 18.0 (SPSS, Inc., Chicago, IL, USA) was used for all statistical analyses.

## Results

### Comparison of demographic and laboratory characteristics of patients between the T2DM and T2DN groups

The average ages of T2DN and T2DM patients were 65 and 62 years, respectively. There were no significant differences in age or sex between the T2DN and T2DM groups (Table 1, all P > 0.05). T2DN patients had increased serum HS, increased urea nitrogen, increased CRP, decreased HGB, increased neutrophils and increased N% compared to T2DM patients (all P < 0.05, Table 1). There were no additional parameters with significant differences between the two groups (all P > 0.05).

**Table 1:** Characteristics of subjects and comparison of demographic and laboratory data of diabetic patients between the T2DN group and the T2DM group by univariate analyses

	Healthy controls (n = 16)	T2DM patients (n = 35)	T2DN patients (n = 35)
Age (years old)	61 ± 9	62 ± 12	65 ± 13
Gender (female)	52%	57%	42%
HS (ng/ml)	1.54 ± 0.60	1.41 ± 0.92	2.17 ± 1.44 ^a,b^
IL-1β (pg/ml)	16.06 ± 6.70	21.35 ± 11.26 ^a^	27.85 ± 14.62 ^a,b^
UARC (mg/g)	4.2(0.08,8.7)	8.1(5.1,11.1) ^a^	58.5(35.9,125.6) ^a,b^
Albumin (g/L)	45.40 ± 2.09	42.92 ± 3.16	41.52 ± 5.57
FGB (mmol/L)	5.32 ± 0.35	8.63 ± 3.36	8.48 ± 3.26
Urea nitrogen (mmol/L)	5.48 ± 1.49	5.33 ± 1.22	6.30 ± 2.52 ^a,b^
Creatinine (umol/L)	69.71 ± 14.12	58.69 ± 12.32	69.71 ± 34.32
Uric acid (umol/L)	360.71 ± 90.16	322.57 ± 85.92	324.93 ± 90.18
Carbon dioxide (mmol/L)	26.47 ± 1.14	26.25 ± 3.06	26.19 ± 2.92
Triglyceride (mmol/L)	1.35(0.92,2.54)	1.43(1.10,2.08)	1.30(0.93,1.95)
Total cholesterol (mmol/L)	4.99(4.50,5.71)	4.34(3.58,4.99)	4.32(3.66,5.07)
HDL (mmol/L)	1.27 ± 0.19	1.10 ± 0.28	1.09 ± 0.28
LDL (mmol/L)	3.20 ± 0.77	2.46 ± 1.01	2.66 ± 0.78
CRP (mg/L)	1.07(0.43,2.83)	0.81(0.45,3.15)	2.03(0.90,6.53) ^a,b^
HbAlc (%)	5.6 ± 0.3	9.2 ± 2.5	9.8 ± 2.9
HGB (g/L)	148 ± 7	141 ± 15	128 ± 19 ^a,b^
Platelet (× 109/L)	215 ± 38	204 ± 51	224 ± 70
Leukocyte (× 109/L)	6.1 ± 1.2	5.9 ± 1.3	6.4 ± 2.1
Neutrophil (× 109/L)	3.5 ± 1.0	3.4 ± 1.0	4.1 ± 1.9 ^a,b^
N%	57 ± 9	56 ± 9	63 ± 10 ^a,b^

*HS* heparin sulphate, *UARC* urinary albumin-to-creatinine ratio, *FGB* fasting blood glucose, *HDL* high-density lipoprotein, *LDL* low-density lipoprotein, *CRP* c-reaction protein, *HbAlc* glycosylated haemoglobin, *HGB* haemoglobin, *N%* neutrophilic granulocyte percentage

^a^ P < 0.05 vs. Healthy controls

^b^ P < 0.05 vs. T2DM

### Comparison of serum IL-1β and HS among the T2DM group, T2DN group and controls

**Fig. 2:**
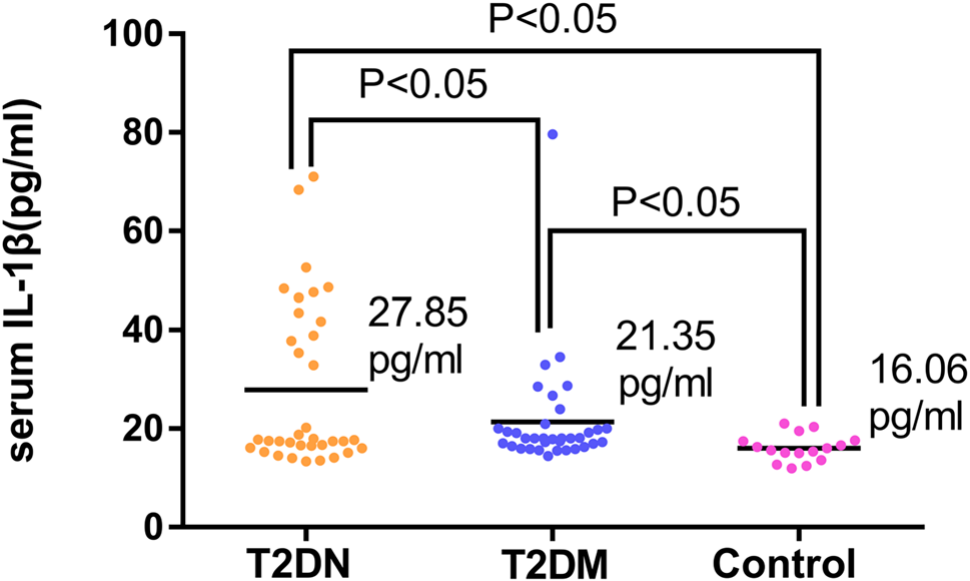
Comparison of serum IL-1β among the T2DM group, the T2DN group and the controls

**Fig. 3:**
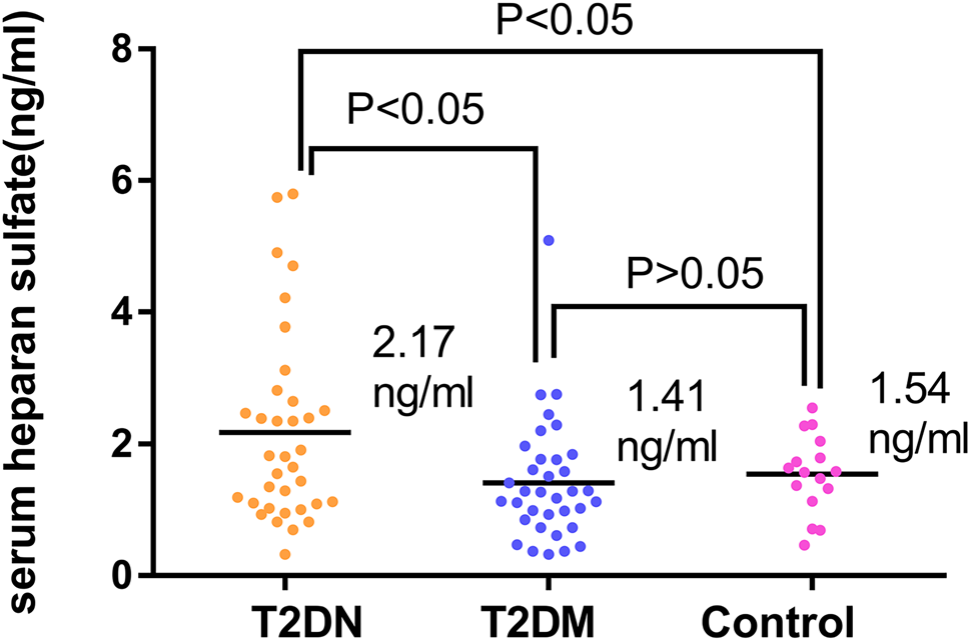
Comparison of serum HS among the T2DM group, the T2DN group and the controls

In this study, the median IL-1β levels steadily increased across the following groups: healthy controls: 16.06 pg/ml; T2DM group: 21.35 pg/ml; and T2DN group: 27.85 pg/ml (ANOVA P < 0.05) (Fig. 2). Serum HS levels were significantly higher in the T2DN group (median 2.17 ng/ml) than in the T2DM group (median 1.41 ng/ml, P < 0.05) or healthy controls (median 1.54 ng/ml, P < 0.05) (Fig. 3). However, there was no significant difference in HS levels between the T2DM group and controls (P > 0.05).

### Logistic regression analyses for identifying factors independently associated with T2DN patients

Variables that were different between the T2DM group and the T2DN group in the univariate analyses, such as HS, urea nitrogen, CRP, HGB, neutrophils and N%, were entered into a logistic regression analysis. The dichotomous dependent variable in the logistic regression analyses was the presence or absence of T2DN. The results showed that HS (B = 0.582) and HGB (B = 1.051) were factors independently associated with T2DN patients (all P < 0.05, Table 2).

**Table 2:** Logistic regression analyses for identifying factors independently associated with T2DN patients

Variable	Exp (B value)	P value	EXP (B value) 95% CI	
			Lower limit	Upper limit
HS	0.582	0.047*	0.342	0.992
CRP	0.977	0.561	0.904	1.056
HGB	1.051	0.029*	1.005	1.099
IL-1β	0.973	0.326	0.922	1.027
FGB	0.997	0.981	0.793	1.254
N%	0.942	0.096	0.878	1.011

*HS* heparin sulphate, *FGB* fasting blood glucose, *CRP* c-reaction protein, *HGB* haemoglobin, *N%* neutrophilic granulocyte percentage, *CI* confidence interval

* P < 0.05

### Prediction of T2DN by HS and HGB

**Fig. 4:**
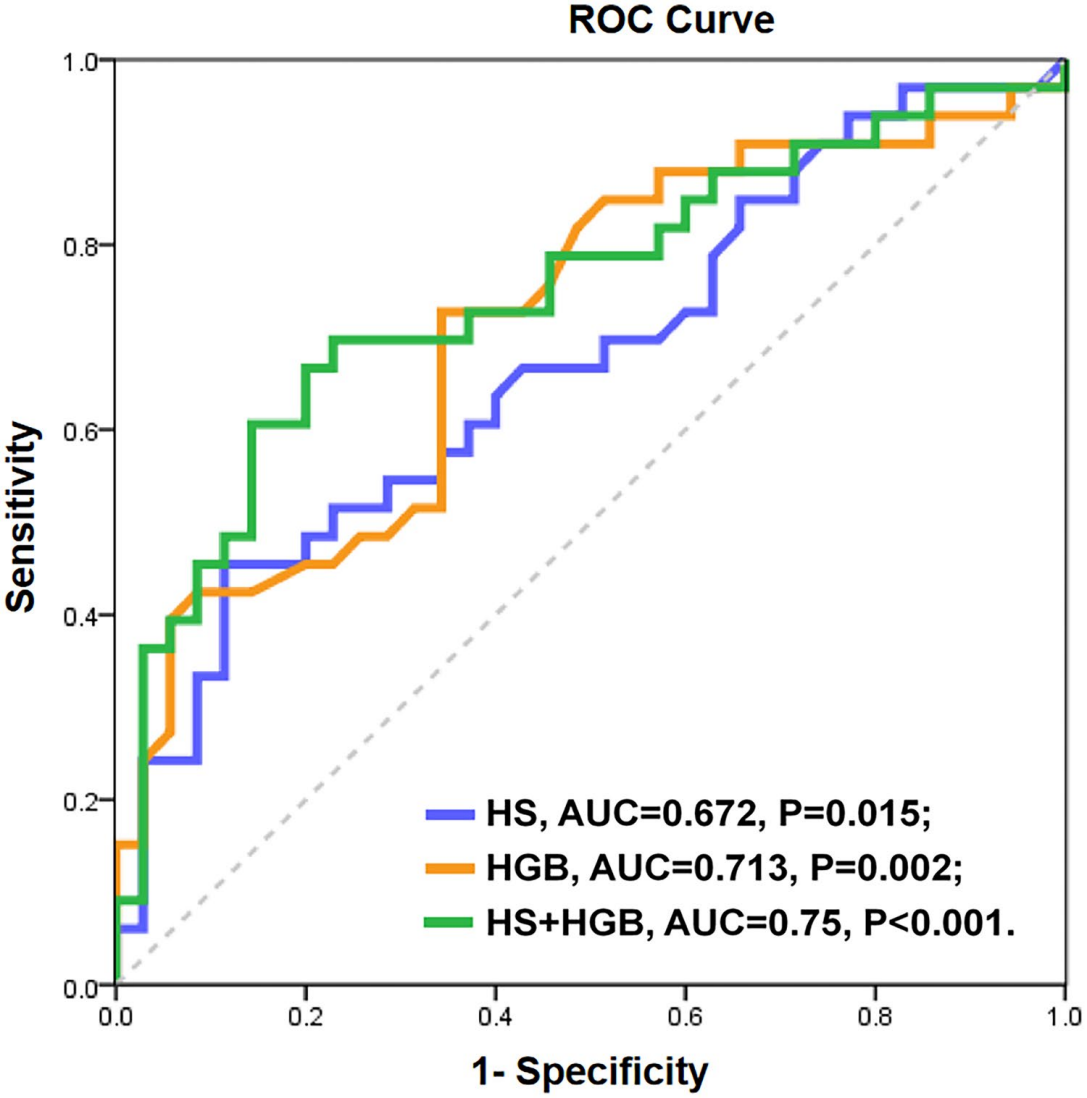
Receiver operating characteristic (ROC) curves for T2DN with each potential predictor. Blue line: HS concentration; orange line: HGB concentration; green line: HS concentration + HGB concentration. Abbreviations: HS, heparin sulphate; HGB, haemoglobin; AUC, area under the ROC curve

To evaluate the discriminative performance of independently associated factors for the prediction of T2DN, ROC curves were constructed (Fig. 4). The AUCs of HS and HGB for the prediction of T2DN were 0.672 (P = 0.015) and 0.713 (P = 0.002), respectively. Both factors achieved statistical significance for T2DN. Furthermore, a combined model of HS and HGB yielded a significant increase in the AUC (0.750, P < 0.001).

### Correlation of serum HS and IL-1β with other variables in the T2DM group and T2DN group

**Fig. 5:**
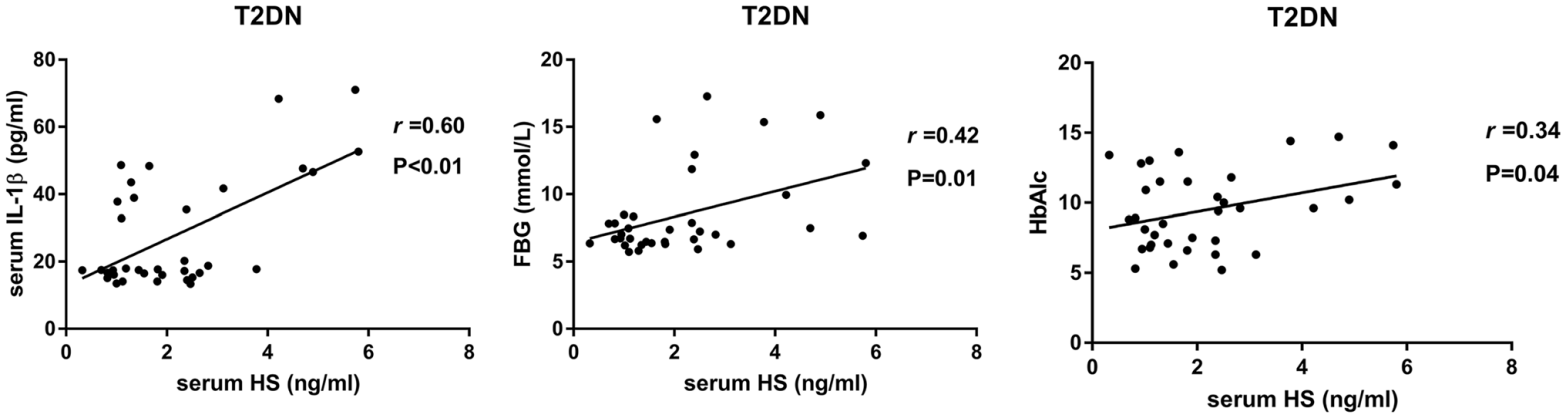
Correlation of serum HS with IL-1β, FBG and HbA1c in T2DN patients. Serum HS was significantly positively correlated with serum IL-1β, FBG and HbA1c in T2DN patients

In T2DM patients, serum HS was significantly positively associated with albumin (P < 0.05, Table 3). There was no significant association between serum IL-1β and HS or other variables in T2DM patients (all P > 0.05). In T2DN patients, serum HS was significantly positively associated with IL-1β, FGB, and HbA1c (P < 0.05, Fig. 5). Serum IL-1β was significantly positively associated with HS, urea nitrogen, and HbA1c (all P < 0.05, Table 3) in T2DN patients.

**Table 3:** Bivariate correlation analyses for the correlation of serum HS and serum IL-1β with other variables in T2DM patients and T2DN patients

	T2DM						T2DN				
	heparan sulfate			IL-1β			heparan sulfate			IL-1β	
	*r*	P value		*r*	P value		*r*	P value		*r*	P value
Age	-0.14	0.43		-0.31	0.07		0.11	0.53		0.27	0.12
HS	/	/		0.19	0.29		/	/		0.60	0.00*
IL-1β	0.19	0.29		/	/		0.60	0.00*		/	/
eGFR	0.00	0.99		0.11	0.52		0.11	0.52		-0.15	0.40
Albumin	0.37	0.03*		0.26	0.14		-0.11	0.53		-0.19	0.28
FBG	0.06	0.75		0.33	0.06		0.42	0.01*		0.13	0.48
Urea nitrogen	-0.05	0.80		-0.20	0.24		0.04	0.81		0.29	0.09
Creatinine	-0.07	0.69		-0.05	0.80		-0.05	0.79		0.15	0.39
Uric acid	-0.08	0.64		-0.03	0.87		-0.17	0.33		0.03	0.89
Carbon dioxide	-0.09	0.62		0.06	0.74		0.04	0.83		-0.12	0.51
Triglyceride	0.22	0.21		-0.06	0.74		0.00	1.00		-0.09	0.61
Total cholesterol	0.17	0.34		-0.02	0.91		-0.01	0.94		-0.19	0.28
HDL	0.09	0.60		-0.03	0.88		0.02	0.94		-0.16	0.38
LDL	-0.26	0.13		-0.02	0.93		-0.11	0.54		-0.15	0.41
CRP	-0.03	0.85		0.16	0.38		0.05	0.79		0.00	0.99
HbAlc	-0.06	0.75		0.23	0.18		0.43	0.02*		0.31	0.09
HGB	0.08	0.63		0.18	0.30		-0.21	0.24		-0.25	0.16
Platelet	0.04	0.83		-0.05	0.78		0.09	0.61		-0.35	0.05
Leukocyte	-0.21	0.23		0.09	0.62		-0.05	0.77		-0.02	0.91
Neutrophil	-0.19	0.28		-0.01	0.95		-0.06	0.74		-0.05	0.77
N%	-0.06	0.74		-0.10	0.58		-0.06	0.76		-0.10	0.57

*HS* heparin sulphate, *FGB* fasting blood glucose *HDL* high-density lipoprotein, *LDL* low-density lipoprotein, *CRP* c-reaction protein, *HbAlc* glycosylated haemoglobin, *HGB* haemoglobin, *N%* neutrophilic granulocyte percentage

*P < 0.05

### Multivariate linear regression analyses for identifying independently associated factors for HS in T2DN patients

Variables that were significantly correlated with HS in T2DN patients (IL-1β, FBG and HbA1c) were entered into multivariate linear regression analyses. The results showed that IL-1β (β = 0.053, P < 0.001) and FBG (β = 0.168, P = 0.008) were factors independently associated with HS in T2DN patients (Table 4).

**Table 4:** Multivariate linear regression analyses for the establishment of factors independently associated with HS in T2DN patients adjusted by HbA1c

Variable	B value		P value	B value 95% CI	
				lower limit	upper limit
IL-1β		0.053	0.000*	0.029	0.077
FGB		0.168	0.008*	0.047	0.289

*FGB* fasting blood glucose, *HbA1c* glycosylated haemoglobin, *CI* confidence interval

*P < 0.05

## Discussion

To the best of our knowledge, this study is the first to show two results. (1) Serum HS levels were significantly and independently correlated with T2DN in all T2D patients; HS (serum HS > 2.32 ng/ml) combined with haemoglobin (HGB < 136 g/L) predicted T2DN with optimal sensitivity (71.2%) and specificity (79%). (2) In T2DN patients, increased serum IL-1β may be an independent factor associated with HS.

Obesity and type 2 diabetes (T2D) represent low-grade chronic inflammatory states. [22, 23] Low-grade inflammation is closely involved in the pathogenesis of T2D and its associated complications. [24] Neutrophils are a major component of the host innate defence against infection and contribute to autoimmune pathogenesis and chronic inflammation. [25] Serum hepcidin levels are increased in chronic inflammation, causing anaemia and inflammation. [26] C-reactive protein (CRP) is a sensitive biomarker of chronic low-grade inflammation. [27] Together, increased neutrophils, reduced haemoglobin and increased CRP are biomarkers of chronic inflammation. In our study, neutrophils and CRP of T2D patients with nephropathy were higher than those without nephropathy, while haemoglobin in T2D patients with nephropathy was lower than that in those without nephropathy. These results suggest that the chronic inflammatory state of DN is more severe than that of diabetes without nephropathy, and this low-grade persistent inflammation may promote DN development and progression.

Generalized EG damage occurs in diabetes [28] and is associated with microalbuminuria (MA). [29] When EG is damaged, its degradation is accompanied by shedding of one or more glycocalyx components into the blood. [30] EG deterioration can be detected using the plasma levels of two well-established biomarkers, syndecan-1 and HS. [31] In our study, serum HS levels were higher in the T2DN group than in the T2DM group and healthy controls, but there were no significant differences between T2DM patients and controls. These results indicate that the EG in T2DN patients is more severely damaged than that of T2DM patients and healthy controls. There was a significant difference between serum HS levels in T2DN and T2DM patients, and HS may be an independently associated factor of T2DN patients in our study. However, Yokoyama *et al*. measured HS in the urine and serum of diabetic patients by two different HS-specific ELISAs (10E4 and 3G10) and did not find significant differences in the serum. [32] In contrast to Yokoyama, we used a similar to but not the same as 3G10 ELISA kit, which detected a degraded serum heparin sulfate fragment in the circulation. Furthermore, Yokoyama divided patients by different urinary albumin excretion rate (AER), in their study, diabetic patients were divided into normoalbuminuria (AER < 12 mg/g. Cr), incipient nephropathy (AER 12 to 220 mg/g. Cr), and clinical nephropathy (AER > 200 mg/g. Cr). In our study, patients were divided into a T2DM group (UARC < 30 mg/g without diabetic retinopathy) and a T2DN group (UARC 30 to 300 mg/g and diabetic retinopathy). As such, the different methods and grouping criteria may produce different results. Deckert *et al*. observed that the de novo synthesis of heparan sulfate was reduced in fibroblasts isolated from diabetes patients with albuminuria but not in those from patients without albuminuria, or healthy control subjects, and formulated a hypothesis that the loss of EG is a prerequisite for developing diabetic nephropathy. [33] This may be the potential mechanism of the research of Yokoyama. The pathogenesis of diabetic nephropathy is very complicated and not yet clear. Our results were not completely contradictory to those of Yokoyama. In the future, large samples and prospective clinical research or basic experimental research are needed for further clarification.

In our study, haemoglobin was another independently associated factor in T2DN patients. The ROC curves showed that the AUC of HS for the prediction of T2DN was 0.67 with good specificity (88.6%) but poor sensitivity (45.4%). When HS was combined with haemoglobin, the AUC increased to 0.75 with optimal sensitivity (71%) and specificity (79%). A 5-year prospective observational study conducted at a diabetes clinic in Australia showed that declining haemoglobin levels were more common in those with higher levels of albuminuria, [34] and this finding was in agreement with our results.

Albuminuria as an outcome of kidney damage is not a specific biomarker for the prediction of DN prior to the onset of this devastating complication. [35] There is an urgent need to determine an easy and accurate way to detect DN prior to its beginning or during the infancy stage so that its progression can be slowed or arrested. Our study revealed that serum HS and/or haemoglobin could be novel biomarkers of T2DN and could help identify potential DN patients among T2D patients early with both diagnostic and prognostic implications. Defining new predictive biomarkers to use alongside UARC during the initial stages of DN would provide a window of opportunity for therapeutic interventions to prevent or delay the onset of the disease, and to improve outcomes.

It is possible that the NLRP3 inflammasome is homeostatic and maintains metabolic balance under normal physiology. However, NLRP3 may be activated by chronic inflammation in diabetes, becoming pathologic and promoting disease. [36] Activation of the NLRP3 inflammasome leads to caspase 1-dependent release of the proinflammatory cytokine IL-1β. [37] Elevated circulating IL-1β may impair islet cell function and induce dysregulation of blood glucose levels, resulting in the progression of T2D and even the development of T2DN, [38, 39] and may play an important role in initiating and sustaining inflammation-induced organ dysfunction in T2D. [40]

In this study, serum IL-1β levels in all T2D patients were significantly higher than those of healthy controls, and serum IL-1β was higher in the T2DN group than in the T2DM group. These results indicate that persistently increased inflammatory factor IL-1β may be involved in the development and progression of T2D and even T2DN. Serum IL-1β was significantly different between T2DN patients and healthy controls, but there was no significant difference between T2DM patients and healthy controls. These results suggest that the increased serum IL-1β occurs earlier than the increased serum HS, which indicates that abnormal inflammasomes appear earlier than damage to the EG during the course of diabetes and may potentially induce damage to the EG. Serum IL-1β and fasting blood glucose (FBG) were independently associated with HS in our T2DN group. As HS may represent a promising biomarker of T2DN, increased serum IL-1β and FBG may predict and promote the progression of DN. These results again indicate that inflammasomes may be associated with and promote EG damage in DN patients, which induces MA in T2D and DN disease courses. Preventing the increase in IL-1β and FBG in patients during the early stage of T2D may be potential therapeutic targets to prevent or delay DN. Advances in basic science and clinical investigations of the mechanism of inflammasomes, the EG and MA in T2D are worth pursuing. Ongoing trials will determine whether the reduction in the NLRP3 inflammasome and/or IL-1β will translate into long-term success in forestalling damage to the EG in T2D and the progression of DN. The anti-inflammatory activities of the NLRP3/IL-1β pathway suggest that it is a promising prospect for DN treatment and provides new ideas for DN treatment.

This study has several limitations. First, it was performed at a single centre, and the sample size was relatively small. Second, the cross-sectional study design precluded the determination of cause and effect. Third, the degree of EG degradation was assessed by measuring serum HS, and this method may not accurately represent current EG integrity or the extent of loss. Fourth, we did not recognize HS by antibodies, which are crucially dependent on specific modification/sulfation motifs in HS. Changes in ELISA signals could be either related to a change in the serum concentration of HS or related to structural changes in HS. Fifth, DN patients with macroalbuminuria and renal insufficiency were not included in this study. Finally, the HS and IL-1β ELISA kits used in this study are available for research use only and are not intended for diagnostic or therapeutic use; these measurement methods are not standardized. These limitations highlight the need for adequately powered RCTs and basic studies to further confirm the findings presented here.

## Conclusions

HS is isolated from porcine intestinal mucosa as a by-product during heparin production. [41] HS is a type of glycosaminoglycan that is attached to the core proteins of proteoglycans, which is a ubiquitous component of the cell surface and in extracellular matrix. [42] In this study, we found that serum HS may be a novel biomarker in the prediction of ongoing/progressive T2DN. Serum IL-1β was associated with HS in T2DN patients, suggesting that inflammasomes may be associated with damage to the endothelial glycocalyx in the T2DN disease course, which is manifested by microalbuminuria. IL-1β maybe a potential therapeutic target of T2DN.

## Supplementary Information

Below is the link to the electronic supplementary material.

Supplementary file1 (DOCX 29.9 KB)

## Data Availability

The data used for the current study are available from the corresponding author upon reasonable request and approval by the principal investigator.
