# Supplementary material for: The association between serum interleukin-1 beta and heparin sulphate in diabetic nephropathy patients
Source: Glycoconj J. 2022 Jan 8;38(6):697–707. doi: 10.1007/s10719-021-10035-7 (PMC8821487; doi:10.1007/s10719-021-10035-7)
Supplement: Supplementary file 1 — Supplementary file1 (DOCX 29.9 KB) [file 10719_2021_10035_MOESM1_ESM.docx]

**Supplementary methods**

For the measurement of IL-1β, 100 µl per well of serum samples and serially diluted IL-1β standard samples were added into 96-well microtiter plates and incubated for 2 hours at room temperature, followed by 100 µl of detective IL-1β antibody per well for 1 hour at room temperature. Horseradish peroxidase-conjugated detective antibody for IL-1β was added (1 hour, room temperature), and the reaction was visualized by the addition of 50 µl tetramethyl benzidine (TMB) reagent for 30 minutes. A solution containing sulphuric acid was used to stop colour development, and the absorbance was measured at 450 nm using an ELISA plate reader (Molecular Devices, SpectraMax M2, USA). Plates were washed five times with washing buffer after each step. As a reference for quantification, a standard curve was established by serial dilutions of IL-1β standard samples (range from 3.5 to 250 pg/ml).

For measurement of HS, 100 µl per well of serum samples and serially diluted HS standard samples were added into 96-well microtiter plates coated with an antibody specific for HS. The HS protein in the samples was captured by the coated antibody after 40 minutes of incubation at room temperature. Biotinylated antibody for HS (100 µl per well) was added to detect the captured HS protein. After 20 minutes of incubation, 100 µl per well of enzyme conjugate was added, followed by TMB reagent. Stop solution was used to stop the reaction. The OD value was measured at 450 nm (Molecular Devices, SpectraMax M2, USA). Plates were washed five times with washing buffer after each step. As a reference for quantification, a standard curve was established by serial dilutions of HS standard samples (the minimum detected concentration was 0.15 ng/mL). A calibration curve was constructed by plotting the absorbance values versus the HS concentrations of the calibrators, and concentrations of unknown samples were determined by using this calibration curve. The inter-assay and intra-assay coefficients of variation were both less than 9.3%.
